# Immune gene expression changes more during a malaria transmission season than between consecutive seasons

**DOI:** 10.1128/spectrum.00960-24

**Published:** 2024-08-20

**Authors:** Kieran Tebben, Salif Yirampo, Drissa Coulibaly, Abdoulaye K. Koné, Matthew B. Laurens, Emily M. Stucke, Ahmadou Dembélé, Youssouf Tolo, Karim Traoré, Amadou Niangaly, Andrea A. Berry, Bourèma Kouriba, Christopher V. Plowe, Ogobara K. Doumbo, Kirsten E. Lyke, Shannon Takala-Harrison, Mahamadou A. Thera, Mark A. Travassos, David Serre

**Affiliations:** 1 Institute for Genome Sciences, University of Maryland School of Medicine, Baltimore, Maryland, USA; 2 Department of Microbiology and Immunology, University of Maryland School of Medicine, Baltimore, Maryland, USA; 3 Malaria Research and Training Center, University of Sciences, Techniques and Technologies, Bamako, Mali; 4 Malaria Research Program, Center for Vaccine Development and Global Health, University of Maryland School of Medicine, Baltimore, Maryland, USA; George Washington University, Washington, DC, USA

**Keywords:** malaria, transcriptomics, RNAseq, *Plasmodium*, immunity, adaptive immunity

## Abstract

*Plasmodium* parasites, the causative organism of malaria, caused over 600,000 deaths in 2022. In Mali, *Plasmodium falciparum* causes the majority of malaria cases and deaths and is transmitted seasonally. Anti-malarial immunity develops slowly over repeated exposures to *P. falciparum* and some aspects of this immunity (e.g., antibody titers) wane during the non-transmission, dry season. Here, we sequenced RNA from 33 pediatric blood samples collected during *P. falciparum* infections at the beginning or end of a transmission season, and characterized the host and parasite gene expression profiles for paired, consecutive infections. We found that human gene expression changes more over the course of one transmission season than between seasons, with signatures of partial development of an adaptive immune response during one transmission season and stability in gene expression during the dry season. Additionally, we found that *P. falciparum* gene expression did not vary with timing during the season and remained stable both across and between seasons, despite varying human immune pressures. Our results provide insights into the dynamics of anti-malarial immune response development over short time frames that could be exploited by future vaccine and prevention efforts.

**IMPORTANCE:**

Our work seeks to understand how the immune response to *Plasmodium falciparum* malaria changes between infections that occur during low and high malaria transmission seasons, and highlights that immune gene expression changes more during the high transmission season. This provides important insight into the dynamics of the anti-malarial immune response that are important to characterize over these short time frames to better understand how to exploit this immune response with future vaccine efforts.

## INTRODUCTION

In 2022, malaria caused over 600,000 deaths worldwide (1). This mortality is primarily caused by *Plasmodium falciparum* infections in children under 5 years old (2), who lack protective immunity. Repeated exposure to malaria leads, first, to development of immunity to severe malaria (typically occurring in early childhood) and, in later childhood, to immunity against clinical symptoms altogether (3, 4). Prior studies have demonstrated development of both a cellular (5) and humoral (6) response to malaria upon repeated exposures. Memory CD4+ T cells specific for *Plasmodium* blood-stage antigens and skewed toward several T cell phenotypes (e.g., Th1, Tfh, Treg) have been observed (7, 8), but their specific role in anti-malarial immune response remains controversial. In mouse models, Th1 cytokine-biased memory cells appear to protect against malaria (9), but further work is needed to characterize these cells in human infections. Memory B cell populations specific for blood-stage antigens have also been shown to develop with age and exposure (10) in a transmission-dependent pattern (11), leading to secretion of *Plasmodium-*specific antibodies (12–15) that contribute to controlling the parasitemia.

However, the development of the adaptive immune memory response may be complicated by inefficient priming of T cells by antigen presenting cells (4), dampening of the immune response by regulatory T cells (16), dysregulation of B and T cells (4, 17) and/or development of atypical memory B cell phenotypes (18–20). In addition, anti-malarial immunity wanes clinically during periods of low exposure (21–24), but the time scale of this waning and its underlying mechanisms remain unclear (25).

While individual acquisition and loss of anti-malarial immunity has been studied longitudinally over the years, the kinetics of development and loss of anti-malarial immunity over both long and short time frames are still incompletely understood. Additionally, the parasite response to changing immune pressure in an infected human during these short periods remains elusive. Previous work has characterized the immune gene expression changes (26) associated with high and low numbers of repeated clinical malaria episodes across an 8-year period (27), while changes in the expression of *P. falciparum* variant surface antigen, PfEMP1, have been linked to changes in immune status (28, 29). Since the *P. falciparum* blood stages are responsible for all clinical symptoms of malaria and since we have access to peripheral blood to examine the human immune response at this stage, studying host and parasite gene expression from infected blood can provide information on how the immune response to peripheral malaria develops over one transmission season, whether this response changes during the dry season, and how the parasite responds to these changes.

In Bandiagara, Mali, malaria transmission is intensely seasonal, with a high transmission wet season from June to December and a low transmission dry season from January to May. Each child 0–14 years of age experiences, on average, 2.2 clinical malaria episodes during the high transmission season, compared to 0.275 during the low transmission season (30). This high seasonality makes Bandiagara an ideal location to study the dynamics of anti-malarial immune responses, related to exposure, over short time frames. Here, we use dual RNA-sequencing analyses of whole blood samples collected during symptomatic *P. falciparum* infections that occurred (i) at the beginning and end of one transmission season and (ii) at the end and beginning of two consecutive transmission seasons, to study the dynamics of the anti-malarial immune response over a short time scale.

## RESULTS

### Dual RNA-sequencing to characterize human and *P. falciparum* gene expression

We extracted and sequenced RNA from whole blood samples collected during 33 symptomatic *P. falciparum* infections from 11 Malian children, aged 1–10 years (Table 1). All samples were collected during a patient-initiated visit due to self-identified malaria symptoms (e.g., fever, headache) and for which *Plasmodium* parasitemia was confirmed by light microscopy (30) (Table 1). The mean parasitemia was 64,338 parasites per microliter of blood (range 225–198,325).

**TABLE 1:** Sample characteristics of the selected participants^*b*^

Early vs late analysis group									
Participant ID	Sex	Ethnicity	Age^*a*^ (years)	Collection date (dd-mm-yyyy)	Collection season	Parasitemia (parasites per µL)	Temp. (°C)	Hgb conc. (g/dL)	Complexity of infection
A	F	Dogon	7	**20-Aug-2010**	Early wet	36,375	39.5	10.6	Monoclonal
				02-Nov-2010	Late wet	225	39.6	10.7	Monoclonal
B	M	Dogon	7	18-Sept-2009	Early wet	56,600	37.5	11.3	Polyclonal
				**17-Sept-2010**	Early wet	83,075	36.1	10.6	Polyclonal
				**17-Nov-2009**	Late wet	97,125	37.5	10.3	Monoclonal
				21-Nov-2010	Late wet	12,450	37.9	9,0	Polyclonal
C	F	Dogon	2	**29-Sept-2010**	Early wet	179,550	39.8	7.7	Monoclonal
				**27-Nov-2010**	Late wet	65,100	38.9	11.6	Monoclonal
D	M	Dogon	5	13-Aug-2012	Early wet	181,800	38.2	11.2	Polyclonal
				09-Nov-2012	Late wet	39,975	38.4	11.6	Polyclonal
E	M	Dogon	5	29-Sept-2010	Early wet	118,500	37.7	11.1	Monoclonal
				**25-Dec-2010**	Late wet	120,400	38.4	10.5	Polyclonal
F	M	Dogon	3	03-Sept-2010	Early wet	37,206	38.4	9.7	Monoclonal
				20-Nov-2010	Late wet	2,100	38.6	6.6	Monoclonal
G	M	Peuhl	5	**17-Sept-2010**	Early wet	91,350	38.8	12.1	Polyclonal
				12-Dec-2010	Late wet	48,325	39.3	11.2	Polyclonal

Late vs early analysis group									
Participant ID	Sex	Ethnicity	Age^*a*^ (years)	Collection date (dd-mm-yyyy)	Collection season	Parasitemia (parasites per µL)	Temp. (°C)	Hgb conc. (g/dL)	Complexity of infection
A	F	Dogon	6	12-Dec-2009	Early wet	900	39.8	9.9	Polyclonal
				**20-Aug-2010**	Late wet	36,375	39.5	10.6	Monoclonal
B	M	Dogon	6	**17-Nov-2009**	Late wet	97,125	37.5	10.3	Monoclonal
				**17-Sept-2010**	Early wet	83,075	36.1	10.6	Polyclonal
C	F	Dogon	1	19-Dec-2009	Late wet	10,800	35.9	9.0	Monoclonal
				**29-Sept-2010**	Early wet	179,550	39.8	7.7	Polyclonal
				**27-Nov-2010**	Late wet	65,100	38.9	11.6	Monoclonal
				12-Aug-2011	Early wet	71,900	38.1	11.7	Polyclonal
E	M	Dogon	5	**25-Dec-2010**	Late wet	120,400	38.4	10.5	Polyclonal
				26-Jul-2011	Early wet	198,325	39.1	12.2	Polyclonal
G	M	Peuhl	4	18-Dec-2009	Late wet	1,125	38.3	9.7	Monoclonal
				**17-Sept-2010**	Early wet	91,350	38.8	12.1	Polyclonal
H	M	Dogon	10	26-Nov-2010	Late wet	47,475	36.2	10.9	Monoclonal
				11-Sept-2011	Early wet	30,475	37.5	10.8	Polyclonal
I	M	Dogon	2	21-Dec-2010	Late wet	186,350	38.3	10.8	Polyclonal
				26-Sept-2011	Early wet	43,800	38.9	10.3	Monoclonal
J	M	Dogon	2	1-Nov-2009	Late wet	43,800	39.4	8.5	Polyclonal
				20-Aug-2010	Early wet	37,550	37.6	6.4	Monoclonal
K	M	Dogon	2	23-Dec-2010	Late wet	15,100	40	9.8	Polyclonal
				30-Sept-2011	Early wet	62,100	39.7	11.9	Monoclonal

^*a*^ Age at first sampled infection.

^*b*^ Collection dates from samples that were included in both analysis groups are indicated in bold.

**Fig 1:**
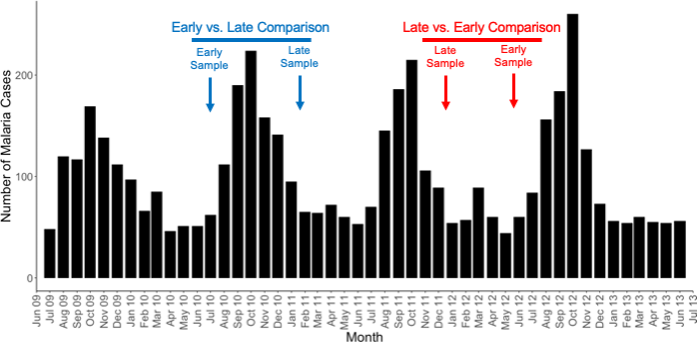
Schematic of the sampling strategy. The black bars show the number of symptomatic malaria cases reported in the entire longitudinal cohort (30) across 4 years. The blue and red arrows illustrate the sampling strategy of paired infections (i.e., from the same child) that would be selected for, respectively, early vs late comparisons (to examine the development of immunity over one transmission season) and late vs early comparisons (to examine the loss of immunity over one dry season).

We included in this analysis blood samples from children that had (i) two *P. falciparum* symptomatic infections in the same transmission season, one at the beginning of the wet season and one at the end (*n* = 8 pairs) (early vs late comparison, in blue on Fig. 1), and/or (ii) two *P. falciparum* symptomatic infections in consecutive years, one at the end of the transmission season of year 1 and one at the beginning of the transmission season of year 2 (*n* = 11 pairs) (late vs early comparison, in red on Fig. 1) (Table 1).

To confirm that *P. falciparum* caused all infections, we first mapped all reads to the genomes of *P. falciparum, Plasmodium vivax, Plasmodium ovale*, and *Plasmodium malariae*, simultaneously, and found that more than 98% of *Plasmodium* reads mapped to the *P. falciparum* genome in each sample (Table S1). We then mapped all reads to the human and *P. falciparum* genomes, simultaneously. We obtained 32–139 million reads mapping to human (49% to 99%) and 0.3 to 50 million reads mapping to *P. falciparum* (0.3% to 50%), allowing robust characterization of host and parasite transcriptomes (Table S1).

To characterize the genetic relationship among parasites from subsequent infections, we genotyped each parasite directly from the RNA-seq reads and reconstructed neighbor-joining trees to assess the relationship between the parasites from all infections (Fig. S1). In both the early vs late (Fig. S1A) and the late vs early (Fig. S1B) comparisons, parasites infecting the same individual at two time points are genetically distinct and appear unrelated.

To understand the main drivers of gene expression in our cohort, we estimated the proportion of variance in human gene expression explained by the patients’ sex, age, the parasitemia of the infection, and by interindividual variation. Consistent with our previous results (31, 32), we found that interindividual variation explained most of the variance in gene expression (patient identifier or PID in Fig. S2) (mean: 14%, range: 0%–96%). To minimize confounding from this variation, we used a paired design, comparing gene expression from samples collected from the same individual, to understand the impact of timing during the transmission season when an infection occurred on gene expression. Interestingly, this variable explained the least variation in gene expression overall.

### Late season symptomatic infections are characterized by a stronger adaptive immune response

We first compared the human gene expression profiles generated from symptomatic infections, from the same child, at the beginning and at the end of one transmission season to investigate potential differences in immune response (*n* = 8 pairs, Table 1). Of 9,181 expressed human genes, 130 genes were significantly differentially expressed (false discovery rate (FDR) < 0.1) between symptomatic infections occurring early versus late in the season, after adjusting for parasitemia (Fig. S3A; Table S2). Interestingly, genes with functions indicative of an adaptive immune response, such as T cell activation [e.g., CCL5 (33), ADA (34)] and T and natural killer (NK) cell granules [e.g., GNLY (35), FGFBP2 (36)], were significantly increased in expression during late season infections. By contrast, genes with functions indicative of an innate immune response, such as pro-inflammatory cytokines [e.g., IL-18 (37)], interferon-stimulated genes [e.g., GBP1 (38), GBP4 (38), GBP5 (38), PARP14 (39)], and regulators of the innate immune system [e.g., CLIC4 (40), LRRK2 (41)], were significantly decreased in expression during late season infections. Overall, this result is consistent with partial development of an adaptive immune response to parasites over repeated exposures throughout the season.

To determine whether these differences in gene expression resulted from changes in white blood cell proportion or true differences in gene regulation, we estimated the relative proportion of each immune cell type using gene expression deconvolution (42) and adjusted our differential expression analyses for the proportion each cell type (collapsing the data first into eight broader cell types to prevent model over-fitting) (Table S2). After adjusting for cell composition, only one gene, myosin light chain 9 (MYL9), remained differentially expressed between early and late season infections, with a higher expression in late season infections (Fig. S3B; Table S2). As a myosin molecule, MYL9 has diverse roles in different cell types, and can interact with the T cell activation marker CD69 to induce inflammation during infections (43). MYL9 has been reported by one study to be expressed during treatment and recovery from malaria (44), and could potentially be involved in promoting an adaptive immune response to infection, although this will require further validation and examination.

**Fig 2:**
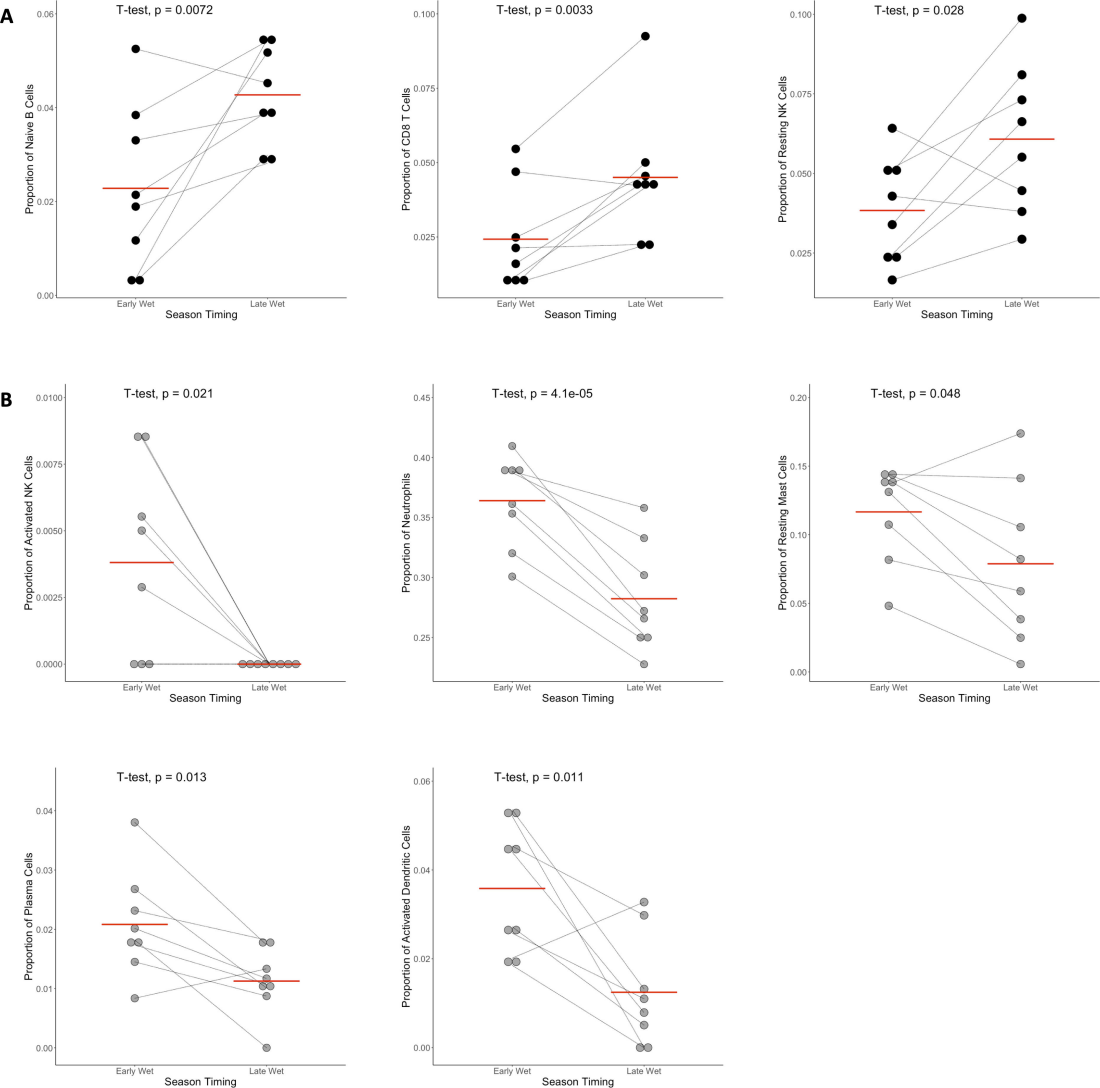
Change in the relative proportion of immune cell types between symptomatic infections occurring early and late in the transmission season. Each panel shows the proportion of one white blood cell (WBC) subset estimated by gene expression deconvolution, with the thin black lines joining estimates from the same individual. (**A**) Cell types that are enriched in late season infections. The panels correspond, from left to right, to naïve B cells, CD8 T cells, and resting NK cells. (**B**) Cell types that are enriched in early season infections. The panels correspond, from left to right and top to bottom, to activated NK cells, neutrophils, resting mast cells, plasma cells, and activated dendritic cells. All comparisons utilize Student’s paired *t*-test with significance was defined as *P* ≤ 0.05. The red bar corresponds to the mean for each group. Note the difference in y-axis scale due to differences in the proportion of each immune cell subtype.

We then examined which immune cell types differed in relative proportion between early and late season symptomatic infections in the same individual. We were able to estimate the proportion of 22 cell types present in the LM22 reference data set (45): naïve B cells, memory B cells, plasma cells, CD8 T cells (total), naïve CD4 T cells, resting memory CD4 T cells, activated memory CD4 T cells, follicular helper T cells, γδT cells, regulatory T cells, resting NK cells, activated NK cells, monocytes, M0 macrophages, M1 macrophages, M2 macrophages, resting dendritic cells, activated dendritic cells, resting mast cells, activated mast cells, eosinophils, and neutrophils. On average, we found that late season infections were characterized by a higher proportion of adaptive immune cells, whereas early season infections were characterized, in general, by a higher proportion of innate immune cells. Specifically, late season infections had proportionally more naïve B cells, CD8 T cells, and resting NK cells in the peripheral blood than early season infections (*P* < 0.03, Fig. 2A). In contrast, early season infections had proportionally more activated NK cells, neutrophils, resting mast cells, plasma cells, and activated dendritic cells in the peripheral blood (*P* < 0.05, Fig. 2B). We found no statistically significant differences in the proportion of memory B cells, CD4 T cells (naïve or memory), T follicular helper cells, γδT cells, regulatory T cells, monocytes, macrophages, resting dendritic cells, activated mast cells, or eosinophils (Fig. S4). These observations could be consistent with a greater role of an innate response in early season infections, while the adaptive immune response dominates late in the transmission season. Of note, the reference data set used for gene expression deconvolution does not contain transcriptional profiles for every immune cell type in the peripheral blood, and more precise techniques such as flow cytometry will be necessary to confirm these results.

### Changes in human gene expression are minimal across the dry season

To begin to understand whether the gene expression associated with developing immunity changes during the dry season (i.e., between transmission seasons), we compared gene expression profiles of 11 pairs of samples from the same children collected during one symptomatic infection at the end of one transmission season and during one symptomatic infection at the beginning of the next transmission season (Fig. 1; Table 1; Table S2). In contrast to gene expression changes observed between infections occurring in the beginning and end of the same season, and despite the larger sample size (11 vs 8), we only identified one gene (MARCO, a macrophage receptor) whose expression was significantly different in this comparison (Fig. S5). This suggests that the acute immune response in the beginning of next season is very similar to that of the end of the previous season, in the absence of interval exposure to infected mosquitoes.

**Fig 3:**
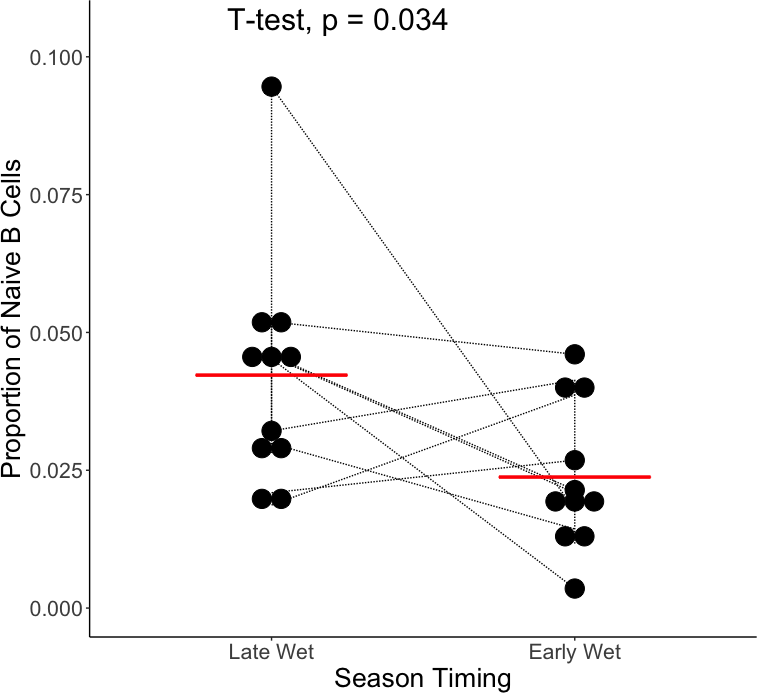
Change in the relative proportion of naïve B cells between symptomatic infections occurring late and early in subsequent transmission seasons. The panel shows the proportion of naïve B cells estimated by gene expression deconvolution, with the thin black lines joining estimates from the same individual, compared with a paired *t*-test. The red bar corresponds to the mean for each group.

Despite the lack of detectable gene expression differences, we analyzed how proportions of immune cells may have changed between transmission seasons (Table S2). We found that infections occurring late in one transmission season had significantly more naïve B cells in the peripheral blood than infections occurring early in the subsequent transmission season (Fig. 3). There were no statistically significant differences in the proportion of any other estimated cell type (Fig. S6). This is consistent with our above findings that late season symptomatic infections are characterized by a more adaptive immune cell signature in the peripheral blood, and the overall immune gene expression signature is similar across the dry season.

### *P. falciparum* gene expression varies minimally over the course of a transmission season or between transmission seasons

Since circulating *P. falciparum* parasites are exposed to the immune system in the blood, we might expect that, as the immune response changes over a transmission season, parasites would vary their gene expression to adapt to changing immune pressures. We first compared the expression of 2,574 *P*. *falciparum* genes from the eight pairs of samples selected from the beginning and end of one transmission season (Table 1). Interestingly, compared to more than 100 differentially expressed human genes between these samples, we identified only nine parasite genes whose expression differed after adjustment for parasitemia (Table S3; Fig. S7). This suggests that, despite changes in host immunity, parasite transcriptional programs in peripheral circulation remain very similar. Of these nine differentially expressed genes, only three had annotated functions and two of the genes, PfPTP3 and PfPTP7, are involved in trafficking the variant surface antigen PfEMP1 to the red blood cell (RBC) surface (46, 47) [the third one, PfFRM2, is involved in daughter merozoite formation (48)]. This observation is interesting since *P. falciparum* has been shown, *in vitro*, to vary PfEMP1 expression in response to environmental changes (29), which likely includes host immune status. Additionally, one study of Kenyan children identified particular PfEMP1 subtypes associated with immune status to severe malaria (28). An important limitation of the current study is that we were unable to compare the expression of specific subtypes of PfEMP1 genes (due to the high sequence homology between PfEMP1 genes and high variability among parasites, it is difficult to map and subtype rigorously PfEMP1s from short-read RNA-seq data). It will be important to follow up on this observation and characterize in future studies whether particular PfEMP1 subtypes are expressed at different times during the transmission season or at different levels of host immunity.

Because of potential changes in host immune pressure over the course of the dry season, we also compared parasite gene expression from 11 pairs of samples selected from the end of one transmission season and the beginning of the next transmission season. Of the 2,483 parasite genes expressed, we did not detect any differentially expressed genes in this analysis (Table S3; Fig. S8). This could support our above results that the host immune response is stable, in the absence of ongoing *P. falciparum* exposure, between transmission seasons and *P. falciparum* parasites are exposed to similar environments during clinical infections. (We also did not observe any differences in the developmental stage composition between early and late samples from the same season, nor between late samples from one season and early samples from the next (Table S3).

## DISCUSSION

Overall, our data suggest that an adaptive immune response to *P. falciparum* partially develops over the course of one transmission season, with evidence from gene expression of activated T and NK cells in late season infections, while we did not detect any changes in immune response during the non-transmission (dry) season. Interestingly, despite this change in host immune gene expression across the transmission season, we did not detect any substantial changes in the *P. falciparum* gene expression.

Prior work has described the development of adaptive immunity to *P. falciparum* over long time frames (4, 6, 49–51) and subsequent waning during periods without consistent parasite exposure (21, 22, 52). We observed an increase in naïve B cells, NK cells, and CD8 T cells in the peripheral blood in late season infections and an adaptive immune gene expression signature, suggesting that an adaptive immune response could incrementally develop within one transmission season. Our observations suggest that the B cell response to *P. falciparum* begins to develop even over a few exposures (but see some limitations below), potentially increasing in proportion in the peripheral circulation after activation and selection in the secondary lymphoid tissues. Because mature plasma cells reside in the bone marrow, their decreased proportion in the blood later in the transmission season is not surprising. Future work such as measuring antibody titers to key antigens and measuring the proportion of B cells with flow cytometry that can differentiate between activated and resting cells is important to provide further insight into the humoral immune response mediated by both of these cell types. NK cells can also produce a memory-like response (53) and mediate efficient killing of infected RBCs in an adaptive-like response (54, 55) in cooperation with *P. falciparum*-specific antibodies developed as part of the humoral response (13, 15, 56, 57). CD8 T cells are implicated in immunity to the liver stages of *P. falciparum* (58), and their enrichment in the blood late season infections could be consistent with an immune response to this stage throughout the transmission season, or an indirect role of these cells in the immune response to blood stages (because CD8 T cells are unable to directly interact with RBCs). Importantly, because we are unable to measure CD8 T cells that are interacting with infected cells in the liver, the proportion of CD8 T cells in the peripheral blood may not be directly related with the immune response to the liver stages and more work is needed to understand their role in blood-stage malaria.

Surprisingly, we did not detect enrichment for memory B and T cells specifically, which could have suggested anti-malarial immune memory development during one transmission season. This could partially be due to the limited resolution of our gene expression deconvolution technique in distinguishing precisely between memory and naïve lymphocyte populations, the atypical, exhausted phenotype of memory lymphocytes that develop after malaria infection and have unique gene expression profiles (17, 18, 20, 59, 60), or a true defect in memory cell generation over few infections. Future work with more high-resolution techniques such as flow cytometry will be necessary to confirm our results and disentangle these possibilities. Taken together, our findings could suggest that despite development of an appropriate adaptive response after malaria exposures (i.e., accumulation of B and T cells late in a season), impairment of the memory response to *P. falciparum* potentially occurs even over a few symptomatic infections during one season, but further work is necessary to confirm these findings and disentangle potential mechanisms.

Interestingly, despite previous evidence of waning antibody titers over the dry season (21), we did not detect any appreciable differences in adaptive immune-related gene expression over the course of one non-transmission season. The lack of differences in gene expression and immune cell composition between late season and subsequent early season infections could suggest that the acute response to infecting parasites remains relatively stable between the end of one season and the beginning of the next, in Mali. While the lack of detectable gene expression differences is not proof of the lack of immunologic differences, it is worth noting here that (i) the sample size for this analysis was slightly larger than for the analysis of the changes during one season and (ii) that the samples spanning one dry season were collected 7–10 months apart (compared to 2–3 months apart for the samples in the same transmission season, where ongoing exposure to *P. falciparum* is occurring). Interestingly, a previous study has reported that parasites can persist as sub-clinical infections through the dry season in Mali, with reportedly little effect on the host immune response (61), and it is possible that these asymptomatic infections could help to maintain immune pressure through the dry season. Of note, Crompton et. al studied changes in antibody titers between transmission seasons in children ages 8–10 years old (21), an age group that is minimally represented in our current study. As such, our work could also highlight age-related differences in the dynamics of the immune responses between transmission seasons and warrants further investigation with larger cohorts.

One important limitation of our current study is its small sample size, which limits overall study power. Individual differences in baseline gene expression, prior malaria exposure and immunity, and number of infections experienced during the malaria season [or sub-microscopic infections experienced during the dry season (61)] likely impact gene expression and may confound our analyses but are difficult to control for due to the sample size. Indeed, prior work has identified age and number of exposures as important determinants in the development of immunity (52, 62, 63). The Peuhl ethnicity has also been associated with genetic protection from malaria (64), and differences in immune gene expression between Dogon and Peuhl individuals could influence our findings. Here, we used paired analyses, comparing samples from the same individual collected during different time points throughout the season, to limit the influence of these individual variations, but future work with larger cohorts, which can better control for these potential confounders, will be essential to confirm and strengthen findings presented here.

Additionally, because our samples were collected when a child presented to clinic with symptoms, all infections included in our analyses were identified and treated at different lengths of time after initial symptom presentation. Specifically, since the initial adaptive immune response takes several weeks to develop, whereas a memory adaptive response or innate response can occur much faster, the interval between infection and diagnosis may influence the gene expression profile. Additionally, treatment of infections could influence the development of a productive adaptive immune response, especially if parasite exposure is very short-lived. We also only included individuals in this study who presented with symptomatic disease and we were unable to precisely measure symptom severity (beyond the lack of severe malaria syndromes), which could introduce an important sampling bias by studying only those individuals who did not yet develop anti-disease immunity to malaria but being unable to directly compare the extent of their clinical disease.

Finally, because we sampled peripheral blood, we were only able to classify the gene expression profiles of parasites and immune cells that are present in circulation. Since mature stages of *P. falciparum* typically sequester in tissues, we may miss important differences in gene expression that occur in non-circulating parasites. Additionally, we could miss important transcriptional differences in immune cells that are not present in circulation, such as those undergoing activation in the secondary lymphoid tissues or directly interacting with sequestered infected red blood cells (iRBCs). While these interactions are important in understanding the complete picture of the anti-malarial immune response, studying the peripheral blood still provides insight into the immune landscape during human malaria.

### Conclusions

In this work, we described the transcriptional profiles of the host and parasite during uncomplicated *P. falciparum* infections occurring at the beginning and end of consecutive transmission seasons. We found that the human immune response changes more over the course of one transmission season than between transmission seasons, despite a lower power (*n* = 8 vs *n* = 11) and a shorter time frame (2–3 months vs 7–10 months). This observation suggests that the immune response to *P. falciparum* changes over a transmission season to adopt an adaptive immune signature later during the transmission season, while it remains relatively stable between transmission seasons. In contrast, we found that *P. falciparum* gene expression varies minimally over this short time scales. Overall, this study contributes new insights into anti-malarial immunity development over repeated exposures during the short time scale of one transmission season. These findings have important implications for understanding the development of protective immunity to malaria that could be exploited by future vaccine and prevention efforts.

## MATERIALS AND METHODS

### Samples

We selected 55 whole blood samples, collected directly in PAXgene blood RNA tubes, from children experiencing a symptomatic uncomplicated malaria episode caused by *Plasmodium falciparum* parasites at the beginning or end of the transmission season in Mali (i.e., June to December). The presence of parasites and the parasite species were initially determined by light microscopy using thick blood smears. All infections were successfully treated with anti-malarial drugs according to the Mali National Malaria Control Programme standards.

### Case definition

Children were classified, by the field clinicians, as experiencing symptomatic uncomplicated malaria if (i) they sought treatment from the study clinic, (ii) they experienced symptoms consistent with malaria (i.e., fever, headache, joint pain, abdominal pain, vomiting, or diarrhea), and (iii) *Plasmodium* parasites were detected, at any density, by thick blood smear, and if they lacked any signs of severe malaria (e.g., coma, seizures, severe anemia) (30).

### Generation of RNA-seq data

We extracted RNA from whole blood using MagMax blood RNA kits (Thermo Fisher). Total RNA was subjected to rRNA depletion and polyA selection (NEB) before preparation of stranded libraries using the NEBNext Ultra II Directional RNA Library Prep Kit (NEB). cDNA libraries were sequenced on an Illumina NovaSeq 6000 to generate ~60–156 million paired-end reads of 75 bp per sample. To confirm that *P. falciparum* was responsible for each malaria episode, we first aligned all reads from each sample using hisat2 v.2.1.0 (65) to a fasta file containing the genomes of all *Plasmodium* species endemic in Mali downloaded from PlasmoDB (66) v.55: *P. falciparum* 3D7*, P. vivax* PvP01*, P. malariae* UG01, and *Plasmodium ovale curtisi* GH01. After ruling out coinfections and misidentification of parasites, we aligned all reads using hisat2 to a fasta file containing the *P. falciparum* 3D7 and human hg38 genomes (i) using default parameters and (ii) using (--max-intronlen 5000). Reads mapping uniquely to the hg38 genome were selected from the BAM files generated with the default parameters. Reads mapping uniquely to the *P. falciparum* genome were selected from the BAM files generated with a maximum intron length of 5,000 bp. PCR duplicates were removed from all files using custom scripts. We then calculated read counts per gene using gene annotations downloaded from PlasmoDB (*P. falciparum* genes) and NCBI (human genes) and the subread featureCounts v.1.6.4 (67).

### Gene expression analysis

Read counts per gene were normalized into counts per million (CPM), separately for human and *P. falciparum* genes. Only human or *P. falciparum* genes that were expressed at least at 10 CPM in >50% of the samples were retained for further analyses. Read counts were normalized via trimmed mean of M-values (TMM) for differential expression analyses. Statistical assessment of differential expression by the time during the season in which a sampled infection occurred was conducted, separately for the human and *P. falciparum* genes, in edgeR (v.3.32.1) (68) using a quasi-likelihood negative binomial generalized model. We used a paired design to make intra-individual comparisons of gene expression between early and late season infections to minimize interindividual effects such as differing levels of developed immunity due to age and exposure history. All models were corrected for the parasitemia of each infection. Adjusted models were corrected for the cell composition of each sample (see below). All gene expression analyses were corrected for multiple testing using FDR (69) (FDR = 0.1).

### Gene expression deconvolution

CIBERSORTx (42) was used to estimate, in each sample, the proportion of (i) human immune cell types and (ii) *Plasmodium* developmental stages. To deconvolute human gene expression profiles, we used as a reference LM22 (45), a validated leukocyte gene signature matrix using 547 genes to differentiate 22 immune subtypes. A custom signature matrix derived from *Plasmodium berghei* scRNA-seq data was used for *P. falciparum* stage deconvolution, using orthologous genes between the two species (70). Relative proportions of each human immune cell type and *P. falciparum* blood stage from each sample are available in Table S4.

### Statistical analysis

All statistical analyses not mentioned above were conducted in R (version 4.0.3). Paired *t*-tests were used to compare cell proportions between groups.

### Complexity of infection

We used samtools (71) mpileup to call the genotype at each sequenced position in all samples directly from the RNA-seq reads. We removed positions within *Plasmodium* multi-gene families due to inaccurate mapping of reads within these regions because of high sequence variability. We then calculated the reference allele frequency (RAF) at each position directly from the resulting files. To determine the complexity of each infection (i.e., monoclonal vs polyclonal), we visualized graphically the distribution of RAF in each sample. Samples with a U-shaped curve, with the RAF for most positions being either 0 or 1, were considered monoclonal. Samples with RAF between 0 and 1, representing a substantial deviation from the U-shaped curve, were considered polyclonal (72).

### Parasite genotyping

To determine whether subsequent infections were caused by the same or different parasites, we used the mpileup files generated directly from the RNA-seq data to identify the parasite genotype(s) at each variable position across all samples using custom scripts. We only considered positions genotyped in more than 12 samples, with a coverage greater than or equal to 50, and variable in at least one sample. Overall, samples with sequences with more than 60% undefined nucleotides (N) were removed from analysis. Six thousand three hundred seventy-eight positions from 14 samples met quality control (QC) criteria for genotyping of the samples collected early and late in the same season. Fifteen thousand two hundred ninety-two positions from 10 samples met QC for genotyping of the samples collected from late in one season and early in the next. We used MEGA11 (73) to reconstruct neighbor-joining trees for each analysis group using the number of nucleotide differences between each pair of samples.

## Data Availability

All sequence data generated in this study are deposited in the Sequence Read Archive under the BioProject PRJNA1111522. Custom scripts are available at https://github.com/tebbenk/seasonality.

## SUPPLEMENTAL MATERIAL

Supplemental figures spectrum.00960-24-s0001.docx Fig. S1-S8.

Table S1 spectrum.00960-24-s0002.xlsx Summary statistics and demographic information for each individual.

Table S2 spectrum.00960-24-s0003.xlsx Human differential expression results.

Table S3 spectrum.00960-24-s0004.xlsx *P. falciparum* differential expression results.

Table S4 spectrum.00960-24-s0005.xlsx Relative proportion of human immune cells and *P. falciparum* developmental stages.

ASM does not own the copyrights to Supplemental Material that may be linked to, or accessed through, an article. The authors have granted ASM a non-exclusive, world-wide license to publish the Supplemental Material files. Please contact the corresponding author directly for reuse.
