# Supplementary material for: Immune gene expression changes more during a malaria transmission season than between consecutive seasons
Source: Microbiol Spectr. 2024 Aug 20;12(10):e00960-24. doi: 10.1128/spectrum.00960-24 (PMC11448414; doi:10.1128/spectrum.00960-24)
Supplement: Supplemental figures — Fig. S1-S8. [file spectrum.00960-24-s0001.docx]

**Supplemental Figures**


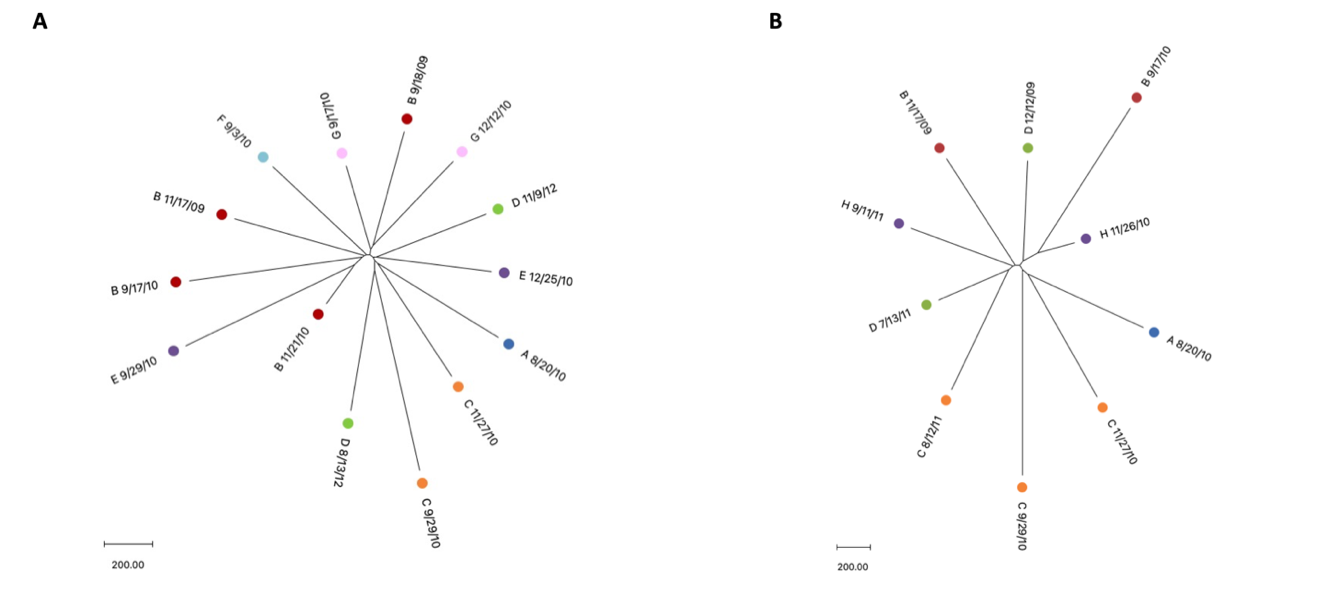


**Supplemental Figure 1**: Genetic relationships among the *P. falciparum* parasites analyzed by RNA-seq. The neighbor-joining trees show the relationships among parasites sampled A) from early and late time infections during the same season and B) from late in one season and early in the next season. The branch lengths are proportional to the number of nucleotide differences between samples. Note that all parasites appear to be roughly equally distant and unrelated, even in consecutive infections from the same individual.


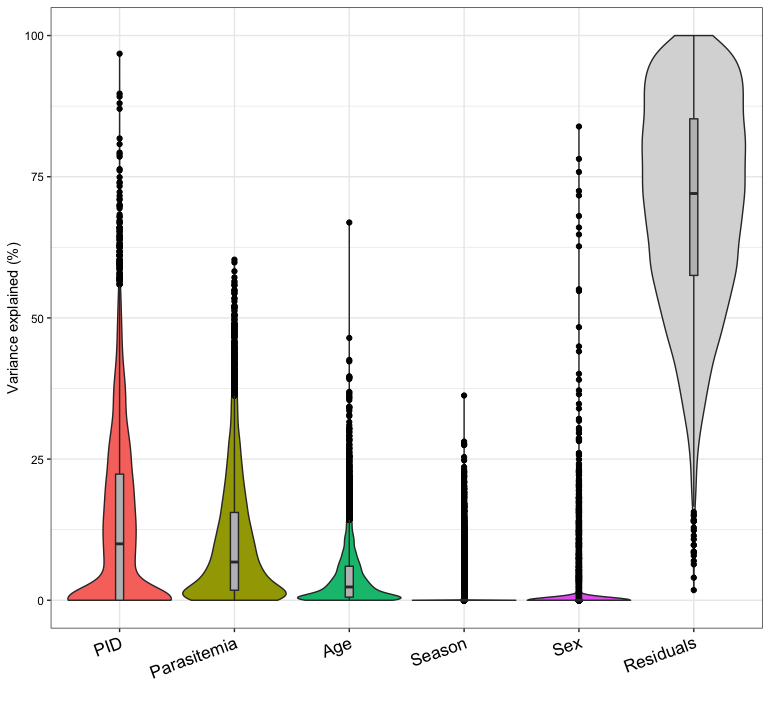


**Supplemental Figure 2: Percentage of the variance in host and parasite gene expression explained by each variable.** Each violin plot shows the percentage of variance explained by one variable for each human gene. Each black dot represents one gene, and the internal boxplot shows the mean variance and 25^th^ and 75^th^ percentiles. “Residuals” indicates the variance in gene expression not explained by any of the included variables.


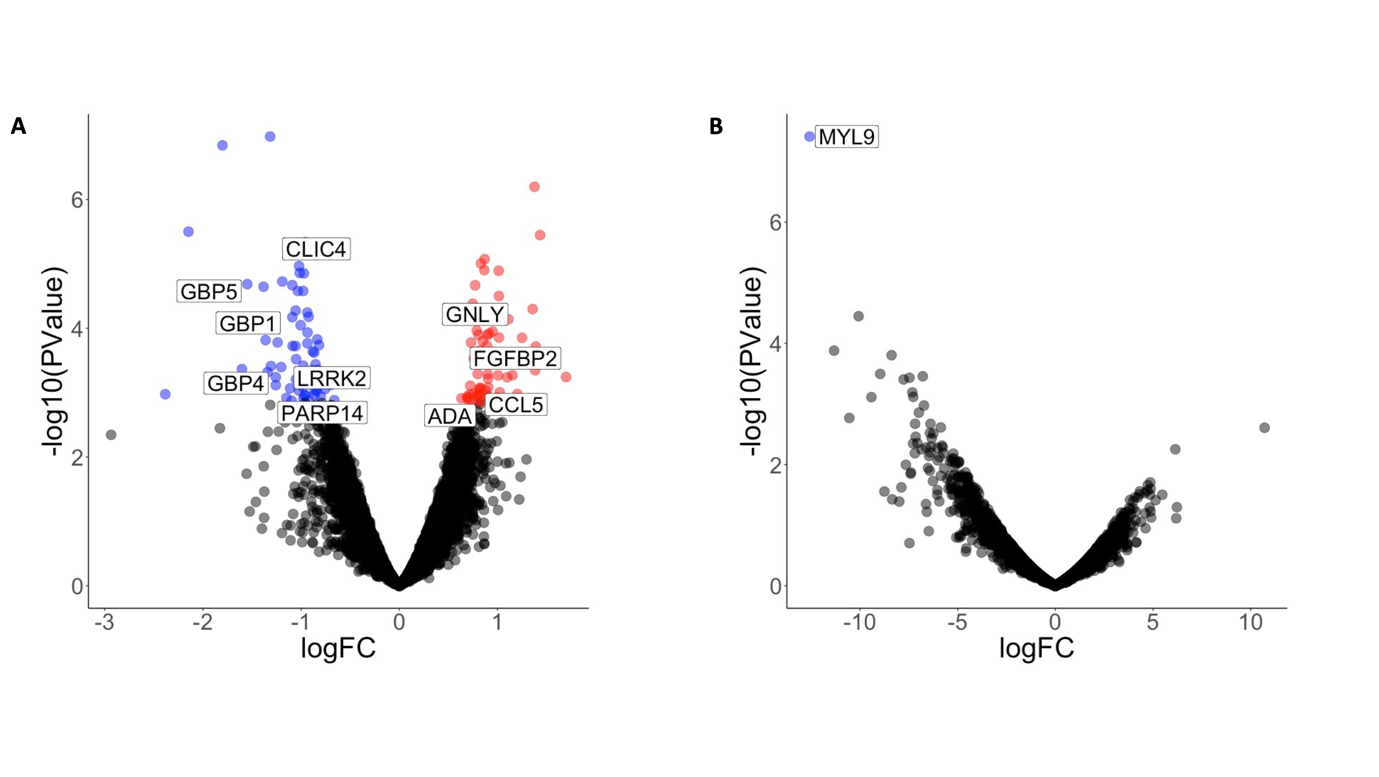


**Supplemental Figure 3: Differences in host gene expression between infections occurring early and late during one transmission season.** Each point represents one gene plotted according to the fold-change and the p-value. Red points represent genes that are more highly expressed in late season infections. Blue points represent genes that are more highly expressed in early season infections. **A)** Before adjustment for cell composition **B)** After adjustment for cell composition.


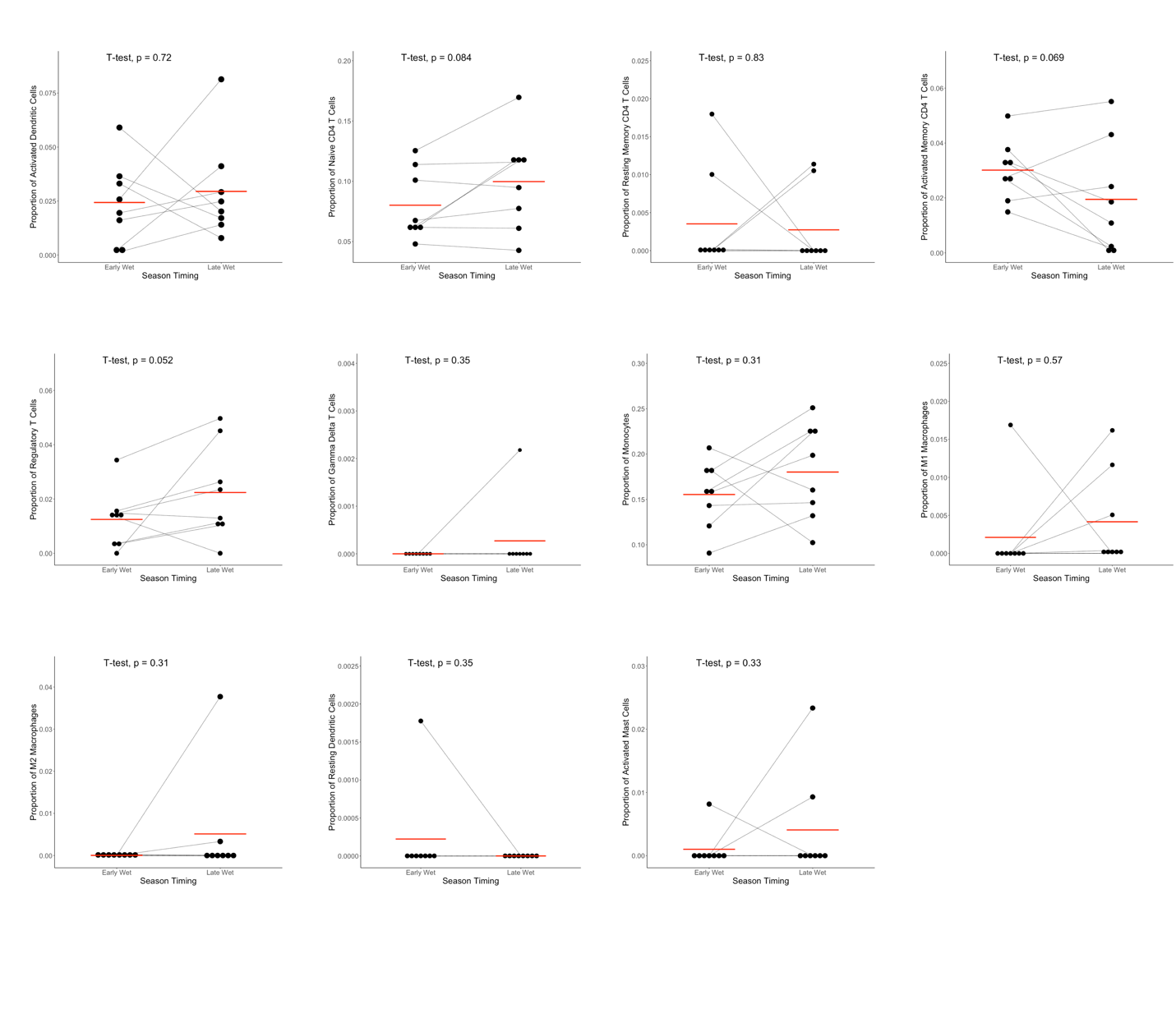


**Supplemental Figure 4:** Relative proportions of immune cell types that did not significantly differ between symptomatic infections occurring early and late in the transmission season. Each panel shows the proportion of one WBC subset estimated by gene expression deconvolution, with the thin black lines joining estimates from the same individual. All comparisons utilize student paired T-test with significance defined as p < 0.05. The red bar corresponds to the mean for each group. Note the difference in y-axis scale due to differences in the proportion of each immune cell subtype; the proportion of eosinophils and follicular helper T cells is not depicted because estimates for all individuals was 0.


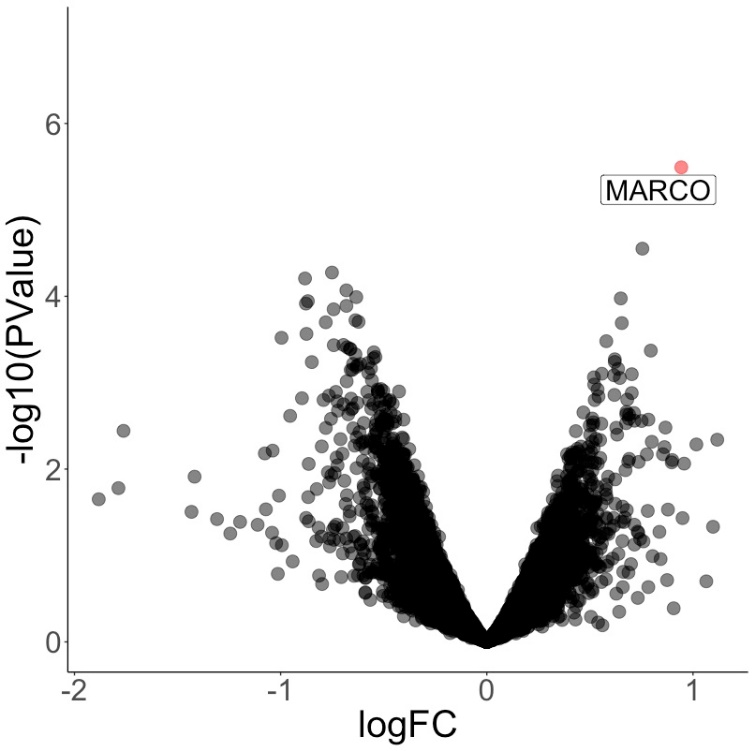


**Supplemental Figure 5: Differences in host gene expression between infections occurring late in one transmission season and early in the next, unadjusted for cell composition.** Each point represents one gene plotted according to the fold-change and the p-value. Red points represent genes that are more highly expressed in late season infections. Blue points represent genes that are more highly expressed in early season infections.


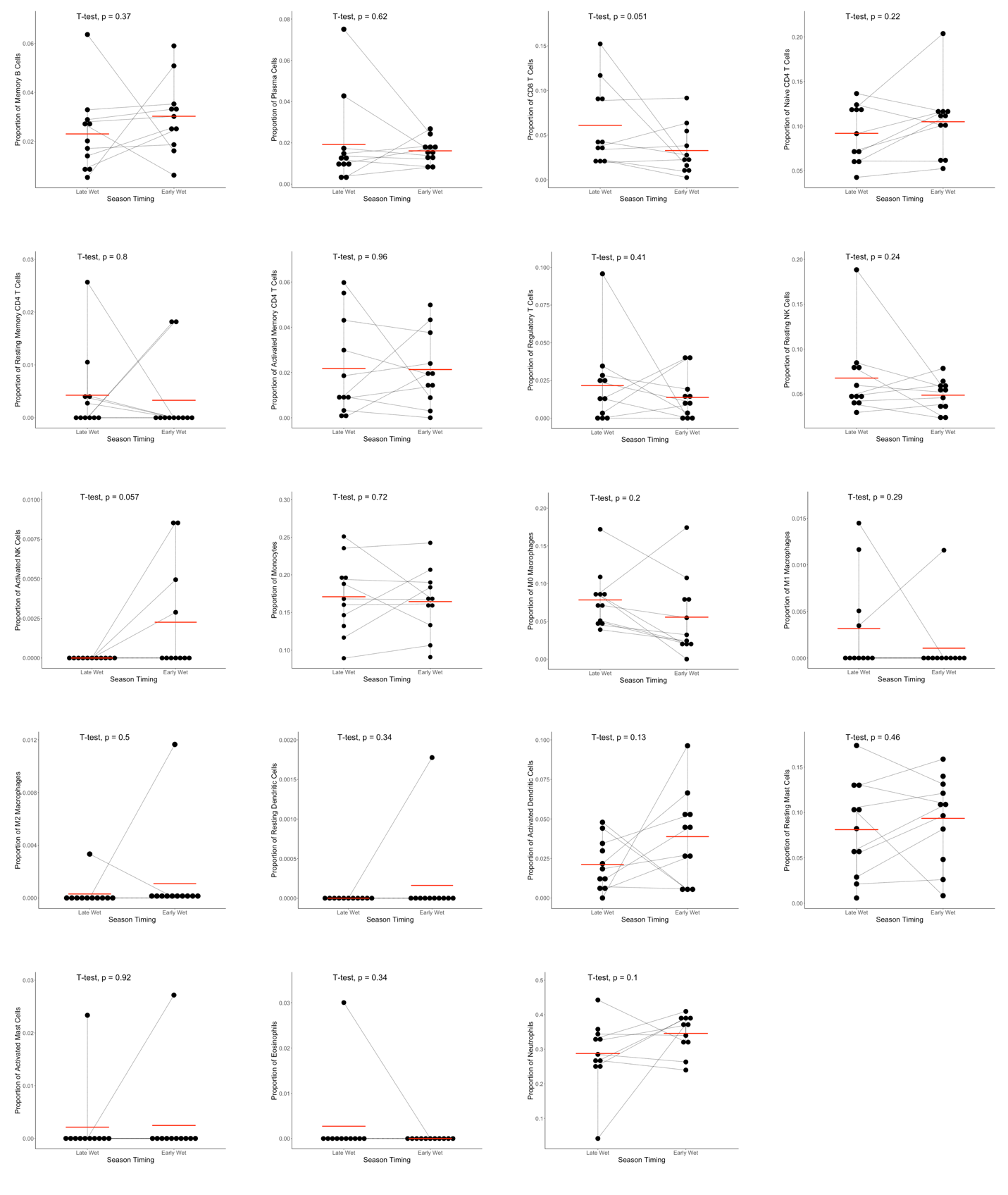


**Supplemental Figure 6: Relative proportions of immune cell types that did not significantly differ between symptomatic infections occurring late in one transmission season and early in the next.** Each panel shows the proportion of one WBC subset estimated by gene expression deconvolution, with the thin black lines joining estimates from the same individual. All comparisons utilize student paired T-test with significance defined as p < 0.05. The red bar corresponds to the mean for each group. Note the difference in y-axis scale due to differences in the proportion of each immune cell subtype; the proportion of gamma delta T cells and follicular helper T cells is not depicted because estimates for all individuals was 0.


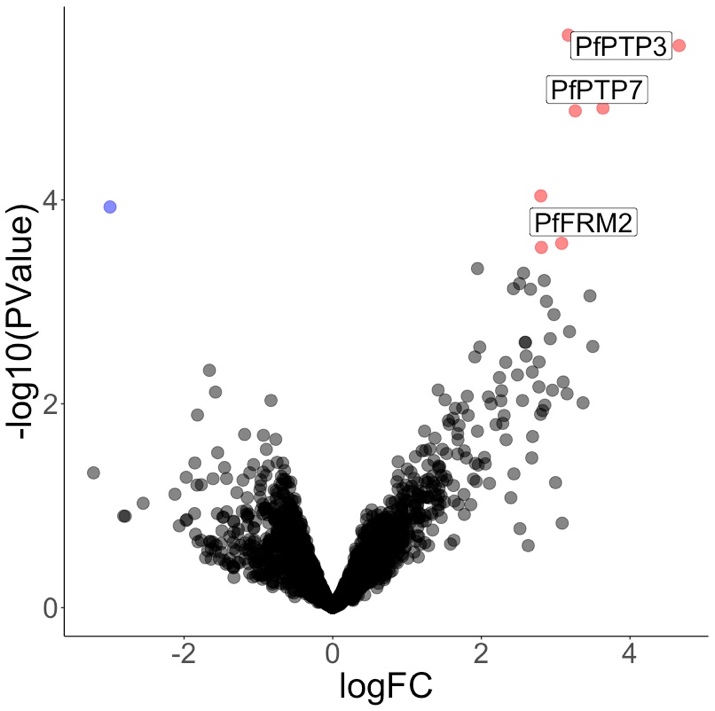


**Supplemental Figure 7: Differences in P. falciparum gene expression between infections occurring early and late during one transmission season, unadjusted for cell composition.** Each point represents one gene plotted according to the fold-change and the p-value. Red points represent genes that are more highly expressed in late season infections. Blue points represent genes that are more highly expressed in early season infections.


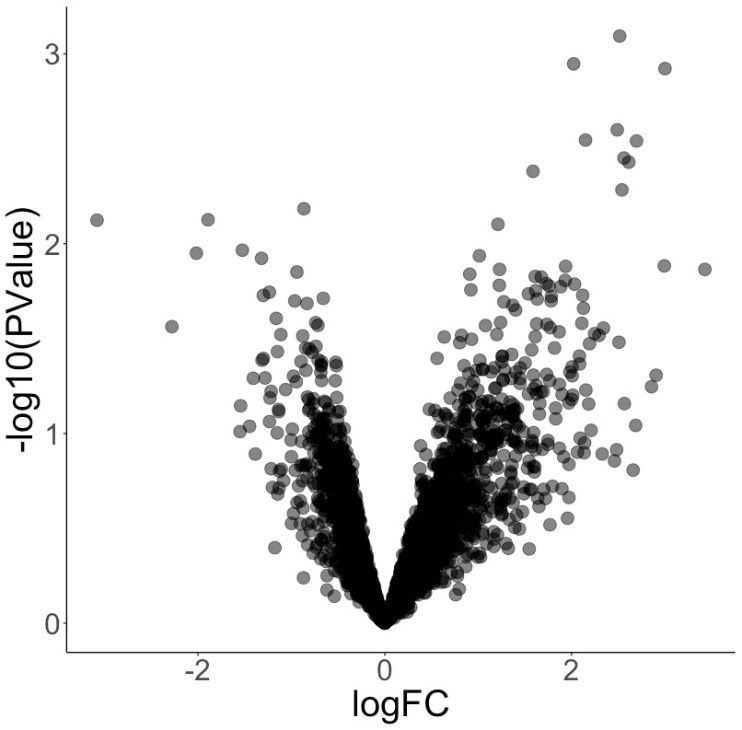


**Supplemental Figure 8: Differences in P. falciparum gene expression between infections occurring late during one transmission season and early in the next, unadjusted for cell composition.** Each point represents one gene plotted according to the fold-change and the p-value. Red points represent genes that are more highly expressed in late season infections. Blue points represent genes that are more highly expressed in early season infections.
